# Ground-Dwelling Arthropod Community Response to Livestock Grazing: Implications for Avian Conservation

**DOI:** 10.1093/ee/nvz074

**Published:** 2019-06-24

**Authors:** Hayes B Goosey, Joseph T Smith, Kevin M O’Neill, David E Naugle

**Affiliations:** 1 Department of Animal and Range Sciences, Montana State University, Bozeman, MT; 2 Wildlife Biology Program, University of Montana, Missoula, MT; 3 Department of Land Resources and Environmental Sciences, Montana State University, Bozeman, MT

**Keywords:** activity-density, arthropod, livestock, rest-rotation, sage-grouse

## Abstract

Terrestrial arthropods are a critical component of rangeland ecosystems that convert primary production into resources for higher trophic levels. During spring and summer, select arthropod taxa are the primary food of breeding prairie birds, of which many are imperiled in North America. Livestock grazing is globally the most widespread rangeland use and can affect arthropod communities directly or indirectly through herbivory. To examine effects of management on arthropod community structure and avian food availability, we studied ground-dwelling arthropods on grazed and ungrazed sagebrush rangelands of central Montana. From 2012 to 2015, samples were taken from lands managed as part of a rest-rotation grazing program and from idle lands where livestock grazing has been absent for over a decade. Bird-food arthropods were twice as prevalent in managed pastures despite the doubling of overall activity-density of arthropods in idle pastures. Activity-density on idled lands was largely driven by a tripling of detritivores and a doubling in predators. Predator community structure was simplified on idled lands, where Lycosid spiders increased by fivefold. In contrast, managed lands supported a more diverse assemblage of ground-dwelling arthropods, which may be particularly beneficial for birds in these landscapes if, for example, diversity promotes temporal stability in this critical food resource. Our results suggest that periodic disturbance may enhance arthropod diversity, and that birds may benefit from livestock grazing with periodic rest or deferment.

Terrestrial arthropods are a dominant component of rangeland macroinvertebrate diversity (Andersen et al. 2004). As key links in rangeland food webs, arthropods occupy many trophic levels and ecological niches, provide ecosystem services (Prather et al. 2013), and convert primary production into resources for higher trophic levels, including species of conservation concern (Gregg and Crawford 2009, Sullins et al. 2018). Thus, changes in arthropod abundance or community composition may have far-reaching effects on rangeland ecosystems. Arthropod community response is therefore a critical component of understanding the influence of land use and management interventions on rangeland ecosystems (Hoffmann 2010).

Particular groups of arthropods are key energetic resources for grassland and shrubland birds, many of which are imperiled in North America (Wiens and Rotenberry 1979, Vickery and Tubaro 1999). For example, spring and summer diets of threatened Mountain Plovers, *Charadrius montanus* (Townsend, Charadriiformes: Charadriidae), in Colorado were composed almost entirely of insects, mostly Coleoptera, Orthoptera, and Hymenoptera (Formicidae) (Baldwin 1971). Diets of nestling grassland-obligate passerines in Saskatchewan were dominated by Lepidopteran larvae, and members of Lepidoptera, Orthoptera, and Coleoptera were preferentially selected (Maher 1979). The importance of these three orders to passerine nestling diets is consistent across a range of North American grassland and shrubland ecosystems (Wiens and Rotenberry 1979). Arthropods are also important components of seasonal diets of declining galliform species, including lesser prairie chickens, *Tympanuchus pallidicinctus* (Ridgway, Galliformes: Phasianidae) and greater sage-grouse, *Centrocercus urophasianus* (Bonaparte, Galliformes: Phasianidae); hereafter, sage-grouse. Early studies reported that 50–60% of the diet of young (1–4 wk) sage-grouse chicks was composed of arthropods, with Orthoptera, Coleoptera, and Hymenoptera (Formicidae) comprising the largest share of food items (Klebenow and Gray 1968). Lesser prairie chickens also feed primarily on Orthoptera and Coleoptera, though Hemiptera were frequently consumed in some areas (Doerr and Guthery 1983). However, these early studies, which employed microhistological analysis of crop contents or feces, likely underestimated intake of soft-bodied arthropods such as Lepidopteran larvae, because these food items are quickly broken down following consumption (Sullins et al. 2018). More recent work has linked survival of young sage-grouse chicks to abundance of Lepidopteran larvae (Gregg and Crawford 2009), and a recent diet study using DNA barcoding revealed the importance of Lepidopterans to lesser prairie chickens (Sullins et al. 2018). Together, avian dietary studies in grassland and shrubland systems indicate a few orders make up the bulk of arthropod food items important for survival.

Livestock grazing is the most widespread land use on rangelands globally (Holechek 2011). Herbivory by livestock affects the composition and physical structure of plant communities (Olff and Ritchie 1998) which in turn, may have positive or negative effects on arthropods (Zhu et al. 2012). Among rangeland taxa, arthropods may be especially sensitive to changes in plant species composition (Borer et al. 2012) and vegetation structural characteristics (O’Neill et al. 2003, 2010). Intensive grazing results in shorter, simpler vegetation structure and may reduce abundance and diversity of arthropods (Kruess and Tscharntke 2002, Debano 2006). Furthermore, because large herbivores can influence arthropods through mechanisms other than vegetation-mediated pathways, grazing is thought to have greater influence on arthropods than plant communities (Pöyry et al. 2006, Rickert et al. 2012).

Grazing by large herbivores can also increase quality or heterogeneity of arthropod habitats relative to ungrazed areas, with positive effects on arthropod abundance or diversity (Rickert et al. 2012, Zhu et al. 2012). Moderate grazing can increase plant aboveground net primary productivity (McNaughton 1979), and thereby increase food resources for phytophagous insects (Tscharntke and Greiler 1995). Under light to moderate stocking rates, livestock graze may selectively induce spatial heterogeneity in vegetation structure (Jerrentrup et al. 2014). Patches of reduced vegetation height or litter depth provide favorable microclimates for certain species (Bestelmeyer and Wiens 2001) and may allow some taxa to increase by suppressing predatory arthropods that depend on tall or complex vegetation structure (Farrell et al. 2015). Seasonal or intermittent grazing can also induce temporal heterogeneity in vegetation, and evidence suggests periodic disturbance such as grazing are important for maintaining high biodiversity in grasslands (Allan et al. 2014). Price (1997) suggested that moderate levels of disturbance produce the greatest arthropod abundance and diversity by keeping competitively dominant species in check. Thus, grazing strategies that incorporate temporal variation in grazing intensity may be an effective tool for maintaining arthropod biodiversity in managed rangelands.

One such management strategy, often advanced for a wide range of conservation goals, is rest-rotation grazing (Hormay 1956). Designed to mimic the natural patterns of herbivory of wild ungulates, rest-rotation grazing involves moving livestock herds through multiple pastures throughout the grazing season while leaving at least one pasture ungrazed to allow for plant growth and reproduction. Rest-rotation grazing has been proposed for arthropod conservation in temperate grasslands (Morris 1991) because the habitat requirements of a broader suite of families is likely to be supported in a landscape comprised of patches diverse in vegetation structure and successional stage. However, despite theoretical conservation benefits, little empirical evidence exists to support the use of rest-rotation grazing systems to enhance rangeland arthropod communities (Dennis et al. 1997; but see Enri et al. 2017).

From 2012 to 2015, we investigated relative abundance and diversity of ground-dwelling arthropods in sagebrush habitats found in grazed and deferred pastures (hereafter collectively called ‘managed’) associated with a rest-rotation grazing system and on adjacent lands that have remained ungrazed for over a decade (hereafter ‘idle’). We focused on ground-dwelling arthropods because of their importance as dietary items of shrubland and grassland birds (Sullins et al. 2018). We hypothesized that ground-dwelling arthropod abundance and diversity would be higher on idle than on managed lands due to greater plant height and ground cover, which could provide more reproductive, thermoregulation, and predator avoidance sites. We also considered the alternative hypothesis that arthropod abundance and diversity may be higher on managed land in response to increased productivity or vegetative structural heterogeneity resulting from periodic and selective grazing. Because arthropod taxa and functional groups exhibit variable responses to grazing and associated changes in plant structure and microclimates (Sjödin et al. 2007), we examined effects of grazing and deferment on select taxa comprising the most important arthropod food items for grassland and shrubland birds in this region. However, to capture the broader ecological implications of the management treatments in our study area, we also tested for treatment effects on ground-dwelling detritivores and predators.

## Methods

### Study Area

Our research area was in central Montana, north of Lavina (46.5176N, 108.0973W) in rolling hill terrain between elevations of 975–1,250 m. The vegetation is intermountain basin big sagebrush steppe (i.e., Wyoming big sagebrush, *Artemisia tridentata ssp*. *Wyomingensis* (Nutt. Asterales: Asteraceae), and silver sagebrush, *Artemisia cana* (Pursh, Asterales: Asteraceae)) with a mixed understory of perennial rhizomatous and caespitose grasses, including western wheatgrass (*Pascopyrum smithii* (Rydb., Poales: Poaceae)), bluebunch wheatgrass (*Pseudoroegneria spicata* (Pursh, Poales: Poaceae)), green needlegrass (*Nassella viridula* (Trin., Poales: Poaceae)), needle-and-thread grass (*Hesperostipa comata* (Trin. and Rupr., Poales: Poaceae)), and blue grama grass (*Bouteloua gracilis* (Kunth, Poales: Poaceae)). Daily temperatures range between 2.88 and 30.88°C. Annual precipitation averages 359 mm with the lowest during our study occurring in 2012 (265 mm) and the greatest in 2014 (485 mm). Commercial agriculture is the dominant land use with about 10% of the land used for crop production.

Concurrent with our study, research on the effect of rest-rotation grazing systems on sage-grouse survival and reproduction was conducted by Smith et al. (2018). Rest-rotation grazing systems, administered by the USDA Natural Resources Conservation Service (NRCS) through their Sage Grouse Initiative (SGI), were voluntarily adopted by several landowners in our study area from 2010 to 2012. Grazing plans were generated individually for each ranch while adhering to the NRCS Montana Prescribed Grazing conservation practice standards. These standards included 1) livestock utilization rates of ≤ 50% of current year’s growth of key forage species, 2) duration of grazing ≤ 45 d, 3) changing timing of grazing by at least 20 d each year, and 4) a contingency plan outlining steps to be taken in exceptional circumstances such as fire or drought. Most landowners elected to incorporate rest into their management plans which provide areas of at least 5% shrub cover, a basic characteristic of sage-grouse nesting habitat, with ~15 mo without grazing on a rotation basis. Ranches participating in SGI rotational grazing systems compromised the two managed land treatments in our study.

### Sampling

**Fig. 1.**
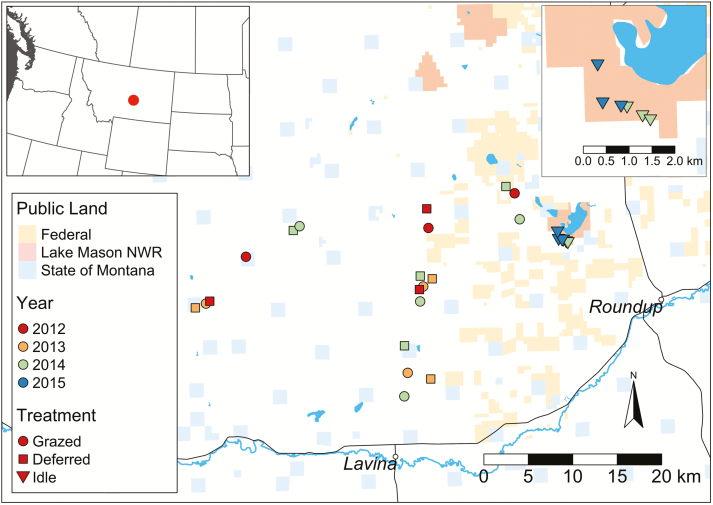
Sampling locations in deferred, grazed, and idle pastures in Musselshell and Golden Valley counties, central Montana, USA, during the 2012–2015 field seasons. The upper right corner inset represents a closer view of idle land sampling locations on the Lake Mason National Wildlife Refuge.

Sampling occurred weekly in three classes of pasture: 1) deferred, 2) grazed, and 3) idle. Grazed pastures had livestock present during sampling. Deferred pastures were in the rest phase and had been excluded from grazing since the spring/summer of the previous year. Idle pastures were located on the Lake Mason National Wildlife Refuge where livestock grazing has been absent for over a decade. Ranch grazing plans are presented in Table 1 and sampling locations are presented in Fig. 1 with sampling dates, temporal replicates, and total activity-density presented in Supp Table 1 (online only). Deferred and grazed pastures were blocked so that both treatments were always sampled on each ranch. Three to four grazed and deferred pastures (experimental unit) were sampled each year, 2012–2014. Idle pasture sampling was conducted on the 1,245 ha Lake Mason National Wildlife Refuge in 2014 and 2015. Grazed and deferred pastures were sampled 2012–2014 while idle land was sampled in 2014 and 2015. Each year, one sampling location was used in each grazed and deferred pasture while three locations were used on idle land. All locations were established in sage-grouse nesting habitat comprised of structural sagebrush with an understory of native grasses and forbs providing food and concealment along with additional insect food resources (Connelly et al. 2011).

**Table 1.** Pasture grazing information obtained from landowners enrolled in the NRCS sage-grouse initiative rest-rotation grazing program during 2012–2014 sampling north of Lavina, MT

Year	Dates grazed	Days grazed	# Head	Animal type	Pasture size
2012	1 June – 16 July	45	81	Cow/calf pair	259
	1 June – 4 July	33	150	Cow/calf pair	260
	6 June – 4 July	45	93	Yearlings	262
2013	9 May – 1 June	24	90	Cow/calf pair	613
	1 May – 5 June	36	225	Cow/calf pair	668
	1 June – 15 July	45	93	Yearlings	405
2014	1 May – 5 June	36	100	Cow/calf pair	445
	21 May – 9 June	20	100	Cow/calf pair	260
	1 May – 15 June	45	100	Yearlings	262
	7 June – 8 July	31	164	Cow/calf pair	520

### Vegetation Characteristics

Weekly and at all sampling locations, we placed a 0.5 m^2^ metal ring (*n* = 10) 2 m apart along a random compass bearing to visually estimated percent bare ground at each location. Also, weekly at an additional 10 random places located no more than 10 m from the pitfall trap transect, the height of the nearest live perennial native grass (excluding inflorescence) and at an additional five random places the height of the nearest live sagebrush (including inflorescence) were measured.

### Arthropod Sampling

Activity-density was estimated weekly with 10 pitfall traps per replicate. Each trap was two, nine-cm diameter; 0.5-liter plastic cups (Solo Cup Company, Lake Forest, IL) stacked together and dug into the ground so that the top cup was flush with the soil surface. Traps were spaced 1 m apart on a linear transect determined by a random compass bearing, were filled one-third full of propylene glycol-based antifreeze (Arctic Ban, Camco Manufacturing Inc., Greensboro, NC), and remained open the entire time between weekly collections. Each trap was fitted with a 25-cm-diameter clear plastic cover suspended on three 10-cm bolts with a minimum 2-cm gap between the soil surface and the cover rim. Traps contents were placed in 11.5 × 23 cm bags (Whirl-Pak, Nasco Inc., Fort Atkinson, WI), the antifreeze was replenished, and samples were returned to the Montana State University for identification according to Triplehorn and Johnson (2005).

Pitfall traps are limited in that insect activity and density are confounded (Topping and Sunderland 1992) and so values obtained from our pitfall trapping were treated as indices of ‘activity-density’ (Kromp 1989). Capture rates may also reflect arthropod habitat selection in addition to differences in population size (Woodcock 2005) because individuals were free to disperse among locations. Despite its limitations, pitfall trapping is a widely used method to sample ground-dwelling arthropods (Spence and Niemelä 1994, Zou et al. 2012). Furthermore, activity-density, as opposed to true density, may be a more meaningful metric of arthropod availability to feeding birds because complex habitats often provide refugia for prey thus reducing capture efficiency (Gotceitas and Colgan 1989). Increased bare ground, for example, positively affects carabid beetle activity-density (Thomas et al. 2006) and is strongly selected by ground-foraging insectivorous birds (Schaub et al. 2010).

### Statistical Analysis

Bare ground (%) and grass and sagebrush heights (cm) were averaged for each sampling location and treatment differences were estimated with Tukey’s Honest Significant Difference Test, assuming normal error distribution using the Proc GLM procedures of SAS (SAS 2008).

Over 4 yr of study, 26 locations were sampled (Supp Table 1 [online only]). Treatment × year interactions were determined using the Proc GLM procedures of SAS (SAS 2008). For mixed model statistical analyses, arthropod counts were summed for each location across sampling weeks into arthropod Families and also into two functional groups of predators and detritivores. An additional group termed ‘Food Arthropods’ was also summed to represent food items of sage-grouse as determined by Klebenow and Gray (1968) and Sullins et al. (2018). **Predators:** Coleoptera: Carabidae, Coccinellidae Histeridae; Araneae: Lycosidae, Gnaphosidae, Philodromidae, Salticidae, Thomisidae, Hahniidae, Linyphiidae, Liocranidae, Dictynidae, Amaurobiidae, and Pisauridae. **Detritivores:** Coleoptera: Tenebrionidae, Scarabaeidae, Dermestidae, Nitidulidae. **Food Arthropods:** Coleoptera: Carabidae, Tenebrionidae, Scarabaeidae, Chrysomelidae, Curculionidae, Coccinellidae; Lepidoptera: Arctiidae, Noctuidae, Saturniidae, Pieridae; Hymenoptera: Formicidae; Orthoptera: Gryllidae, Acrididae, Tettigoniidae.

Summed sampling location counts were analyzed with a generalized linear mixed model using the Proc GLIMMIX procedures of SAS version 9.2 with sampling year as a random variable. Summed counts were fit to a negative binomial distribution and standardized using an offset to account for the variable number of sampling weeks among sites and years with the marginal likelihood approximated using Laplace’s method. Including vegetation characteristics as covariates did not uniformly improve AIC values for all comparisons so for model simplicity and consistency, we dropped these variables from further consideration. Differences among treatment least squared means were calculated using the least significant difference (LSD) test. Contrasts, using the same model, were also estimated for functional and food arthropod groups to compare managed (i.e., the weighted average of deferred and grazed data) and idle pastures to assess land management strategies in our study that do and do not incorporate livestock grazing (Proc GLIMMIX, SAS 2008).

Simpson’s 1-D Family-level diversity was estimated for each replicate using Past v. 3.19 (Hammer et al. 2001) and these values were subjected to the Proc MIXED, LSD procedures of SAS (SAS 2008) with fixed treatment and random year assuming a normal error distribution to test for treatment differences.

## Results

### Vegetation

Bare ground was highest in grazed pastures (*F* = 18.48; df = 2,7; *P* = 0.0049). Grass height tended to differ among treatments (*F* = 4.84; df = 2,7; *P* = 0.0674) with the shortest grass in grazed pastures. Sagebrush height did not differ among treatments (*F* = 0.43; df = 2,7; *P* = 0.6713; Table 2).

**Table 2.** Mean bare ground, grass height, and sagebrush height ± SEM from Deferred, Grazed, and Idle locations during 2012–2015 field seasons and the study average of the same metrics collected north of Lavina, MT

		Sample location metrics		
Year	Location	Bareground (%)	Grass (cm)	Sagebrush (cm)
2012	Deferred	29.12 ± 4.40	19.98 ± 2.00	36.15 ± 3.84
	Grazed	36.10 ± 8.63	16.99 ± 1.59	38.82 ± 4.37
	Idle^*a*^	---	---	---
2013	Deferred	36.04 ± 4.69	24.76 ± 3.70	42.41 ± 4.64
	Grazed	53.38 ± 8.01	17.98 ± 3.56	32.88 ± 5.74
	Idle^*a*^	---	---	---
2014	Deferred	37.38 ± 10.16	21.34 ± 6.78	34.11 ± 6.78
	Grazed	42.32 ± 6.32	19.04 ± 1.58	34.06 ± 3.43
	Idle	10.83 ± 4.18	22.89 ± 3.69	37.68 ± 6.08
2015	Idle	8.33 ± 3.48	21.18 ± 2.43	37.40 ± 5.26
Study Average^*b*^				
	Deferred	34.18 ± 2.56a	22.02 ± 1.42	37.56 ± 2.49
	Grazed	43.93 ± 5.05a	18.08 ± 0.59	35.25 ± 1.81
	Idle	9.58 ± 1.25b	22.04 ± 0.85	37.54 ± 0.14

^*a*^Not sampled during the 2012 and 2013 field seasons.

^*b*^Study average means in columns followed by different letter groupings statistically differ (α = 0.05); Tukey’s Honest Significant Difference Test (Proc GLM, SAS Institute 2008) where all comparisons df = 2,20.

### Arthropods

Over 37,000 arthropod specimens were collected and identified from 54 families during 4 yr of study. Diversity analyses included counts from all 54 Families while functional group, food group, and Family-level analyses were conducted on counts from select taxa of 32 Families. Analysis of variance indicated no treatment × year interactions for total arthropods (*F* = 2.10; df = 2,25; *P* = 0.155), food arthropods (*F* = 2.18; df = 2,25; *P* = 0.142), predators (*F* = 0.59; df=2,25; *P* = 0.566), or detritivores (*F* = 0.01; df = 2,25; *P* = 0.993), nor were interactions detected at the Family level (all Family-level interactions were: *F* ≥ 3.33; df = 2,25; *P* ≥ 0.059); therefore, data were combined across years for analyses.

### Weekly Activity-Density

**Fig. 2.**
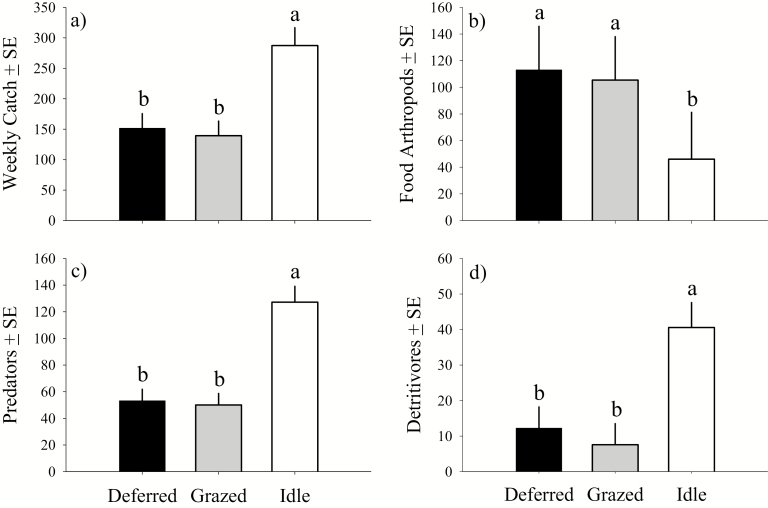
Deferred, grazed, and idle pasture activity-density for a) all arthropods, b) food arthropods, c) predators, and d) detritivores where bars represent normal distribution weekly catch least squared means and error bars represent the standard error. Deferred and grazed pastures are associated with livestock grazing while idle land has not be grazed by livestock in over a decade. Sampling was conducted during the 2012–2015 field seasons north of Lavina, MT. Bars with different letters differ (α = 0.05); A generalized linear mixed model with random year and a negative binomial distribution were fit to count data offset by sampling weeks; LSD (Proc GLIMMIX, SAS Institute 2008) where all comparisons df = 2,20.

Contrast analyses indicated that total arthropod activity-density was twice as high on idle compared to managed land (*F* = 69.55; df = 1,20; *P* < 0.001). Arthropod pasture level activity-density was greatest in idle compared to grazed and deferred, which did not differ from each other (Fig. 2a; Table 3).

**Table 3.** Treatment least squared means ± SE of 2012–2015 weekly catch, food arthropods, and the functional groups of predators and detritivores collected from pitfall traps located in deferred, grazed, and idle pastures north of Lavina, MT

	Treatment			Defer vs. graze		Defer vs. idle		Graze vs. idle	
	Deferred	Grazed	Idle	*t*	*P*	*t*	*P*	*t*	*P*
Weekly catch	151.46 ± 24.86b	139.25 ± 24.86b	287.25 ± 30.15a	1.16	0.261	5.07	<0.001	4.36	<0.001
Predators^*a*^	53.11 ± 9.01b	50.02 ± 9.01b	127.20 ± 12.28a	0.66	0.514	7.24	<0.001	7.85	<0.001
Detritivores^*b*^	12.24 ± 6.09b	7.56 ± 6.09b	40.55 ± 7.16a	1.33	0.199	5.72	<0.001	6.70	<0.001
Food Arthropods^*c*^	113.14 ± 33.03a	105.38 ± 33.03a	46.05 ± 35.48b	0.91	0.373	3.70	0.001	4.40	<0.001

Least squared means in rows followed by different letter groupings statistically differ (α = 0.05); LSD (Proc GLIMMIX, SAS Institute 2008) where all comparisons df=2,20.

^*a*^Predator functional group is the summed total of Coleoptera: Carabidae, Coccinellidae, Histeridae; Araneae: Lycosidae, Dictynidae, Amaurobiidae, and Pisauridae.

^*b*^Detritivores is the summed total of Coleoptera: Tenebrionidae, Scarabaeidae, Dermestidae, Nitidulidae.

^*c*^Food Arthropods is the summed total of Coleoptera: Carabidae, Tenebrionidae, Scarabaeidae, Chrysomelidae, Curculionidae, Coccinellidae; Lepidoptera: Arctiidae, Noctuidae, Saturniidae, Pieridae; Hymenoptera: Formicidae; Orthoptera: Gryllidae, Acrididae, Tettigoniidae.

### Food Arthropods

Contrast analyses indicated that food arthropod activity-density was twice as high on managed compared to idle land (*F* = 53.60; df = 1,20; *P* < 0.001). Pasture level activity-density was also lowest in idle compared to deferred and grazed, which did not differ (Fig. 2b; Table 3).

Activity-density of Coleoptera represented the largest portion of food items in deferred (33.7%), grazed (26.4%), and idle (29.5%) pastures (Table 4). Ranked second was Lepidoptera larvae in deferred (25.5%) and grazed (18.5%) and Orthoptera in idle pastures (27.7%) (Table 5). Ranked third was Orthoptera activity-density in deferred (8.8%) and grazed (12.5%) and Lepidoptera in idle pastures (2.1%) (Table 5). Among Coleoptera, food arthropod activity-density of Carabidae and Chrysomelidae did not differ among deferred, grazed, and idle pastures (Table 4). Tenebrionidae, Coccinellidae, and Curculionidae activity-density differed with the largest weekly catch recorded in deferred and grazed pastures. Scarabaeidae activity-density was greatest in idle when compared to deferred and grazed pastures (Table 4). Gryllidae, Acrididae, and Tettigoniidae treatment activity-density did not differ but Formicidae was greatest in idle compared to deferred and grazed pastures (Table 4).

**Table 4.** Arthropod family least squared means ± SE of weekly activity-density during 2012–2015 field seasons from pitfall traps located in deferred, grazed, and idle pastures north of Lavina, MT

	Treatment			Defer vs. graze		Defer vs. idle		Graze vs. idle	
	Deferred	Grazed	Idle	*t*	*P*	*t*	*P*	*t*	*P*
*Coleoptera*									
Carabidae	12.25 ± 5.25	12.28 ± 5.25	0.55 ± 6.11	0.10	0.921	1.24	0.228	1.32	0.203
Tenebrionidae	11.12 ± 2.86a	6.29 ± 2.86ab	0.56 ± 3.26b	2.00	0.060	2.69	0.019	1.35	0.191
Scarabaeidae	3.25 ± 2.18b	2.64 ± 2.18b	14.33 ± 2.12a	1.09	0.287	4.70	<0.001	5.66	<0.001
Chrysomelidae	2.09 ± 0.79	1.64 ± 0.79	−0.37 ± 0.89	0.63	0.533	0.24	0.816	0.12	0.908
Curculionidae	4.90 ± 1.73a	3.46 ± 1.73a	−0.99 ± 2.12b	0.90	0.378	2.47	0.023	2.19	0.041
Coccinellidae	1.57 ± 0.69	1.43 ± 0.69	−0.48 ± 0.70	0.05	0.963	0.01	0.989	0.01	0.989
Histeridae	0.50 ± 8.98b	0.65 ± 8.98b	39.16 ± 9.72a	0.79	0.443	6.70	<0.001	5.77	<0.001
Dermestidae	0.42 ± 0.60b	0.16 ± 0.60b	5.50 ± 0.65a	1.26	0.227	5.38	<0.001	5.86	<0.001
Meloidae	0.35 ± 2.77b	1.84 ± 2.77b	21.08 ± 2.99a	1.28	0.216	6.66	<0.001	6.17	<0.001
Silphidae	1.62 ± 3.33b	2.86 ± 3.33b	46.00 ± 4.30a	1.67	0.110	6.75	<0.001	5.13	<0.001
Nitidulidae	−1.46 ± 3.96b	−0.14 ± 3.96b	12.84 ± 4.00a	0.69	0.501	3.35	0.004	2.80	0.014
Elateridae	15.57 ± 6.33	4.68 ± 6.32	5.00 ± 7.56	1.35	0.191	0.66	0.5193	1.72	0.102
Melyridae	1.78 ± 3.77	7.96 ± 3.77	−1.14 ± 3.90	2.47	0.026	0.01	0.994	0.01	0.993
*Lepidoptera*									
Noctuidae	4.18 ± 2.63a	5.24 ± 2.63a	−2.46 ± 2.88b	0.60	0.555	2.74	0.012	3.12	0.005
Arctiidae	24.49 + 4.79a	12.84 ± 4.79a	−0.82 ± 6.03b	0.83	0.416	3.14	0.005	2.48	0.022
Saturniidae	0.12 ± 0.10	0.36 ± 0.10	0.01 ± 0.13	1.68	0.109	0.00	0.998	0.00	0.998
Pieridae	0.01 ± 0.69	1.08 ± 0.69	1.11 ± 0.89	0.00	0.998	0.00	0.998	3.02	0.007
*Hymenoptera*									
Formicidae	39.96 ± 15.49b	47.00 ± 15.49b	32.62 ± 17.66a	0.17	0.863	2.82	0.011	3.14	0.005
*Orthoptera*									
Gryllidae	5.49 ± 4.40	8.82 ± 4.41	2.93 ± 5.10	0.85	0.403	0.86	0.400	1.50	0.142
Acrididae	4.58 ± 2.67	4.37 ± 2.67	7.96 ± 3.26	0.24	0.811	0.56	0.560	0.76	0.455
Tettigoniidae	0.04 ± 0.03	0.03 ± 0.03	0.01 ± 0.04	0.26	0.7984	0.00	0.998	0.00	0.998
*Araneae*									
Lycosidae	15.44 ± 4.72b	14.76 ± 4.72b	76.84 ± 5.99a	0.11	0.913	8.56	<0.001	8.92	<0.001
Gnaphosidae	15.76 ± 3.45a	15.24 ± 3.45a	5.73 ± 4.07b	0.24	0.759	2.43	0.023	2.29	0.032
Philodromidae	4.49 ± 0.91	3.04 ± 0.91	1.74 ± 1.11	1.62	0.114	1.01	0.325	2.03	0.056
Salticidae	2.83 ± 0.55a	1.95 ± 0.55ab	0.68 ± 0.67b	1.26	0.220	2.30	0.031	1.50	0.146
Thomisidae	1.52 ± 0.40	1.72 ± 0.40	−0.09 ± 0.51	0.24	0.816	0.01	0.993	0.01	0.993
Hahniidae	0.58 ± 0.42	0.68 ± 0.42	−0.15 ± 0.53	0.19	0.848	0.00	0.996	0.00	0.996
Linyphiidae	0.87 ± 0.34	0.94 ± 0.34	0.19 + 0.34	0.13	0.900	0.00	0.998	0.00	0.998
Liocranidae	0.89 ± 0.28	0.93 ± 0.28	0.13 ± 0.29	0.27	0.794	0.00	0.999	0.00	0.997
Dictynidae	0.21 ± 0.16	0.25 ± 0.16	0.08 ± 0.17	0.20	0.847	0.00	0.998	0.00	0.998
Amaurobiidae	0.32 ± 0.14	0.25 ± 0.10	0.01 ± 0.10	0.42	0.677	0.00	0.995	0.00	0.995
Pisauridae	0.28 ± 0.34	0.47 ± 0.34	0.29 ± 0.34	0.99	0.336	0.01	0.995	0.01	0.995

Least squared means in rows followed by different letter groupings statistically differ (α = 0.05); A generalized linear mixed model with a random year effect and a negative binomial error distribution was fit to count data offset by sampling weeks; LSD (Proc GLIMMIX, SAS Institute 2008) where all comparisons df = 2,20.

**Table 5.** Least squared means ± SE of Simpson’s 1-D for Total Diversity and Food Arthropod Diversity calculated from 2012 to 2015 pitfall traps located in deferred, grazed, and idle pastures north of Lavina, MT

	Simpson’s 1-D^*a*^			Defer vs. graze		Defer vs. idle		Graze vs. idle	
	Deferred	Grazed	Idle	*t*	*P*	*t*	*P*	*t*	*P*
Total Diversity	0.88 ± 0.02ab	0.90 ± 0.02a	0.81 ± 0.03b	0.87	0.392	1.96	0.062	2.72	0.012
Food Arthropod Diversity	0.71 ± 0.03a	0.70 ± 0.03a	0.59 ± 0.04b	0.23	0.821	2.07	0.050	1.87	0.075

Least squared means in rows followed by different letter groupings statistically differ (α = 0.05); A generalized linear mixed model with a random year effect and a negative binomial error distribution was fit to count data offset by sampling weeks; LSD (Proc MIXED, SAS Institute 2008) where all comparisons df=2,20.

^*a*^Simpson’s 1-D calculated from 54 families.

### Predators

Contrast analyses indicated that predator activity-density was twice as high on idle compared to managed land (*F* = 63.17; df = 1,20; *P* < 0.001) and that treatment activity-density was greatest in idle compared to grazed and deferred pastures, which did not differ (Fig. 2c; Table 3). Predator activity-density in idle, compared to grazed and deferred pastures, was dominated by an approximate fivefold increase in Lycosidae. A total of 11 Araneae Families were captured in deferred and grazed compared to only four in Idle pastures (Table 4).

### Detritivores

Contrast analyses indicated that Detritivore activity-density was three times greater on idle compared to managed land (*F* = 43.40; df = 1,20; *P* < 0.001) and that pasture level activity-density was greatest in idle compared to deferred and grazed, which did not differ (Fig. 2d; Table 3).

Captures of Tenebrionidae, Nitidulidae, and Scarabaeidae represented over 95% of total activity-density in deferred and grazed pastures. Nitidulidae, Histeridae, and Scarabaeidae represented approximately 94% of the activity-density on idle land (Table 4). Activity-density of dung-feeding Scarabaeidae was greatest in idle compared to grazed and deferred pastures (Table 4) and was dominated by four dung feeding beetles, *Rhyssemus germanus* (L., Coleoptera: Scarabaeidae)*, Canthon pilularius* (L., Coleoptera: Scarabaeidae)*, Onthophagus nuchicornis* (L., Coleoptera: Scarabaeidae), and *Aphodius distinctus* (Müller, Coleoptera: Scarabaeidae). Scarabaeidae activity-density was 4.4 and 5.4 times greater in idle than in deferred and grazed pastures, respectively (Table 4).

### Family-Level Diversity

Total activity-density diversity, measured with Simpson’s 1-D, was greater in grazed than idle pastures (*F* = 2.72; df = 2,20; *P* = 0.012) but did not differ from deferred which did not differ from idle. Food arthropods were most diverse in deferred and grazed, and least diverse in idle pastures (Table 5).

## Discussion

Differences in arthropod community structure among treatments mirrored patterns in vegetation structure, with the largest contrasts between managed and idle land. Although total arthropod activity-density was greatest on idle land, this pattern masked important variation in responses of arthropod taxa to long-term absence of grazing. Greater activity-density on idle land was driven by a tripling of detritivores and a doubling of predators, primarily Lycosid spiders. In contrast, food arthropod activity density was two times greater and Family-level diversity slightly greater on managed land. Arthropod diversity may be particularly important to birds if it promotes temporal stability in this critical food resource (Sullins et al. 2018). In total, we observed 9 of 14 food arthropod Families at their lowest activity-density on idle land, but low statistical power likely limited our ability to detect significant reductions of some families. Interestingly, most of these Families achieved their highest activity-density in deferred pastures, suggesting they may benefit from structural changes in vegetation (Pöyry et al. 2006, Fritch et al. 2017) afforded by periodic rest from grazing, for example, via increased reproduction or overwintering survival. However, long-term absence of grazing appears to have altered the structure of the arthropod community such that these benefits were negated. Notably, the activity-density of lepidopteran larvae, which are especially important prey for a variety of birds including greater sage-grouse (Gregg and Crawford 2009), was lowest on idle land. Other studies have reported that low (Lyons et al. 2017) and intermediate levels (Kaltas et al. 2013) of grazing can increase both abundance and species richness of carabid beetles, an avian food source.

In contrast to other studies (Jepson-Innes and Bock 1989, O’Neill et al. 2010), our results suggest that Orthopteran activity-density is unchanged in grazed areas. However, our sampling method may have obscured negative effects of grazing on Orthopteran activity-density if reduced vegetation structure on managed land positively biased capture rates relative to the more structurally complex idle treatment (Schirmel et al. 2009). Activity-density of Formicidae was greatest on idle land, and may contrast with other studies, suggesting ants respond more strongly to differences in soil and vegetation type (not measured in our study) than grazing (Hoffmann 2010). Though we did not include Araneae among avian food focal taxa, their importance to avian diets is debatable and may vary by Family (Gunnarsson 2007). In several studies, spiders were fed to nestlings of grassland and shrubland passerines (Wiens and Rotenberry 1979, Petersen and Best 1986); however, these studies do not report the particular Families represented in diets. Lycosids are sit-and-pursue predators, which quickly stalk and ambush prey (Dondale 2005) in the lower vegetative canopy and on the ground (Schmitz and Suttle 2001), which may make Lycosids a particularly challenging prey for birds. That Lycosids did not appear in the diets of lesser prairie chickens despite the common appearance of other Araneae Families suggests they may not be important foods for precocial young of grouse (Sullins et al. 2018). Regardless of whether Araneae are excluded outright from food arthropods or only non-Lycosid spiders are included, the relative trends in activity-density of food arthropods among treatments remains the same.

The most notable consequence of long-term idling was reduced bare ground, likely from increased litter cover, as grass and shrub cover were similar among treatments. Differences among treatments are consistent with Smith et al. (2018), who observed increased litter cover and reduced bare ground on the Lake Mason National Wildlife Refuge. These effects of grazing exclusion on vegetation structure are generally consistent with findings in other North American grassland or shrubland ecosystems where researchers have documented greater litter depth (Naeth et al. 1991) cover (Adler and Lauenroth 2000) or biomass (Willms et al. 2002, Farrell et al. 2015) and reduced bare ground cover (Naeth et al. 1991, Yeo 2005) in ungrazed compared to grazed areas (but see Davies et al. 2018).

The reduction of bare ground from litter accumulation is reflected in higher activity-density of predators on idle land. This finding agrees with previous work showing predatory arthropods respond positively to vegetation and detritus (Langellotto and Denno 2004) and are more abundant without grazing (Farrell et al. 2015, Fritch et al. 2017). Predator families differed in their response to treatments; the fivefold higher activity-density of Lycosidae on idle land was accompanied by a reduction in most other predatory Families. Notably, 9 of 13 predatory Families were never captured on idle land. North American Lycosids are, with the exception of *Sosippus* spp., (Araneae: Lycosidae), nonweb building predators that stalk and ambush prey and may therefore benefit from elevated levels of detritus, which provides abundant cover. Increased habitat complexity is also thought to decrease intraguild predation and cannibalism (Denno et al. 2005). Although increases in Lycosid spider activity-density may indicate reduced cannibalism, our observation of reduced Family-level diversity on idle land suggests Lycosids, where they achieve extreme dominance, may exert strong top-down effects on arthropod communities including exclusion of other predatory taxa, perhaps through intraguild predation.

The accumulation of litter is also reflected in higher activity-density of all detritivores except Tenebrionidae on idle land. The opposite response of Tenebrionidae suggests that long-term grazing exclusion does not consistently benefit species belonging to this family. Similarly, Newbold et al. (2014) reported from the short grass steppe higher species richness and greater abundances of certain Tenebrionidae species in long-term grazing exclosures, but also reported that some species increased with grazing.

Unexpectedly, we recorded lower activity-density of Scarabaeidae, most of which were dung beetles, in subfamilies Aphodiinae (Leach) and Scarabaeinae (Larreille), on managed land despite presumably greater dung food resources. Moderate livestock grazing typically benefits dung beetle populations (Lobo et al. 2006, Verdu et al. 2007). Dung beetles opportunistically feed on detritus (Holter et al. 2002), which was abundant on idle land; however, detritus would only benefit adults as their young are provisioned with dung (Scholtz et al. 2009a). An alternative explanation for reduced dung beetle activity-density on managed land is the treatment of livestock with veterinary parasiticides. Residues of these parasiticides pass from the animal to the pasture via dung and lethal to sublethal effects can manifest in dung colonizing species (Wall and Benyon 2012).

We hypothesize high plant detritus may also explain high activity-density of Dermestidae and Nitidulidae on idle land, whereas high activity-density of Histeridae may be a response to abundant eggs and larvae. Dermestidae, Nitidulidae, and Histeridae are diverse families of beetles associated with plant detritus, souring plant fluids, dung, and carrion. Dermestidae and Nitidulidae primarily consume these items while Histeridae mainly prey upon the smaller insects and eggs living in these materials (van Emden 2013). Silphidae are primarily necrophagous with adults of some species feeding on plant detritus or dung (Anderson and Peck 1985). Approximately 98% of Silphidae in our study were identified as *Nicrophorus spp*. (Coleoptera: Silphidae), which are carrion obligates of small mammal carcasses. Therefore, higher activity-density of Silphidae suggests small mammals may be more abundant on idle land. Small mammals, especially microtine rodents, are often positively associated with vegetation biomass and commonly respond negatively to large mammal grazing (Grant et al. 1982, Warren and Frisina 2013). Given that Dermestidae and Nitidulidae are common catches in carrion beetle traps (Shubeck 1976) we also speculate their presence on idle land could be in part due to a higher small mammal population providing an additional food source.

Pitfall trapping may be subject to bias if treatments differ in structural complexity (Melbourne 1999); however, inference in that study was limited to ants (Hymenoptera: Formicidae). We documented differences in bare ground among treatments, suggesting greater cover of bare ground on managed lands may have been partly responsible for higher activity-density of some taxa. However, total activity-density was generally highest in deferred pastures, which had less bare ground than grazed pastures. We therefore doubt that the confounding effect of habitat structure on arthropod activity-density substantially affected our inference. Despite its limitations, pitfall trapping remains a very suitable method to sample mobile ground-dwelling arthropods (Taboada et al. 2010, Oxbrough et al. 2012), many of which are food items for birds.

Pitfall trapping may not accurately reflect abundance of certain vegetation-dwelling taxa such as grasshoppers (Orthoptera: Acrididae) across gradients in vegetation structure (Schirmel et al. 2009). However, our use of pitfall traps reflects our objective to characterize relative availability of arthropods for consumption by sage-grouse chicks, which are incapable of flight. Given this physical constraint, only those arthropods located on or very near to the soil surface are available for consumption. Thus, although we cannot infer treatment effects on abundance of grasshoppers, activity-density measured by pitfall trapping remains a useful index of food availability for ground-foraging birds. Furthermore, Schmitz (2005) reports from a caged field study on behavioral changes in *Melanoplus* spp. grasshoppers, a genus common to our study area, in response to various spider hunting modes. The presence of a canopy-dwelling nursery web spider (*Pisaurina* spp., Araneae: Pisauridae) caused grasshopper prey to move to locations closer to or on the soil surface. In contrast, when a ground-dwelling Lycosid spider (*Rabidosa* spp., Araneae: Lycosidae) was introduced, grasshoppers moved from the soil surface to higher vegetation. As the most abundant predator on idle lands by a large margin, Lycosid spiders may elucidate an additional indirect reduction of available chick food on the soil surface regardless of whether a reduction in absolute abundance occurred.

**Fig. 3.**
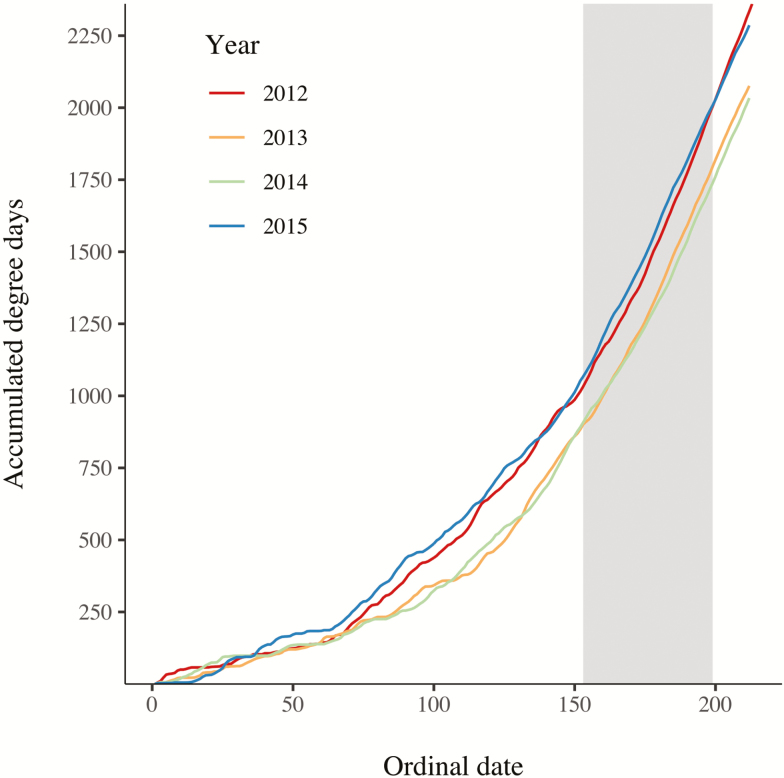
Yearly degree-day accumulations (Y-axis) calculated by the single-sine method (Coop 2002) from weather station HORSE THIEF HORM8 RAWS (46.4256N; 108.6742W; elevation 1,061 m) using 0° C lower and 35° C upper thresholds versus ordinal date (X-axis). The vertical shaded bar represents the range of ordinal dates where our sample occurred during the 2012–2015 field seasons and displays virtual analogues accumulations of degree-days between 2015 where Idle was sampled but not Managed and 2012 where Managed was sampled but not Idle and also between 2013 and 2014. This indicates that the inferences we have drawn from these data are appropriate in relation to the discussed arthropod taxa.

Sampling of Lake Mason National Wildlife Refuge was added opportunistically during the study as new project partners brought new questions and resources. The idle treatment therefore lacked the replication and dispersion of our grazed and deferred treatments and was sampled in a different, but overlapping, set of years. We included these samples in spite of these caveats because it was the only nearby, ecologically similar land where livestock grazing had been absent for a long period and therefore presented a unique opportunity to measure the effects of livestock exclusion on arthropods. Sampling in different years has the potential to confound our inference regarding treatment differences if nonoverlapping years differed with respect to degree-day accumulation (Coop 2002), the primary environmental factor determining arthropod emergence and activity. However, degree-day accumulation in 2015 (idle but no managed land sampling) and 2012 (managed but no idle land sampling) were virtual analogues of each other (Fig. 3) as were 2013 and 2014 when all treatments were sampled. This alleviated our initial concern that treatment and year effects could be strongly confounded. Furthermore, our inclusion of a random effect for year should help ensure that comparisons reflect treatment differences rather than annual effects. Nevertheless, additional replication would help clarify the generality of our findings with respect to the effect of grazing exclusion on arthropod communities in sagebrush rangelands.

Periodic deferment or rest are commonly prescribed to achieve desired conservation outcomes on rangelands and our findings suggest several arthropod taxa comprising important food resources for shrubland and grassland birds may benefit from this practice. In this grazing-adapted ecosystem, however, long-term absence of grazing or other disturbance dramatically altered the structure of the vegetative and arthropod communities, ultimately resulting in reduced availability of the arthropod taxa most important to shrubland and grassland avifauna.

Livestock grazing, by periodically removing aboveground plant biomass and reducing litter accumulation, may indirectly suppresses predatory arthropods, such as Lycosidae, thus numerically releasing populations of prey taxa (Price et al. 2011) some of which are critical food items for birds. Thus, when viewed from the perspective of energy availability for higher trophic levels, managed grazing may serve a valuable conservation function. Dung-feeding Scarabaeidae were a notable exception and if their reduced activity-density is a response to livestock parasiticide use, we would expect that mitigation of this source of mortality or morbidity would further increase this food source on managed lands. Conservation of dung beetles due to their contributions to functioning ecosystems needs to become more of a focal point for land management due to their innate characteristics as bio-indicators (Scholtz et al. 2009b) and their quick response to vegetative changes (Tocco et al. 2013). Given the importance of all arthropod taxa, not only as critical avian food but also as vital components of healthy ecosystems, further studies into the effects of land use on food web processes are needed for conservation of native species and native landscapes.

## Supplementary Material

nvz074_suppl_Supplementary_Table_1
