# Supplementary material for: Ground-Dwelling Arthropod Community Response to Livestock Grazing: Implications for Avian Conservation
Source: Environ Entomol. 2019 Jun 24;48(4):856–66. doi: 10.1093/ee/nvz074 (PMC6681935; doi:10.1093/ee/nvz074)
Supplement: nvz074_suppl_Supplementary_Table_1 [file nvz074_suppl_supplementary_table_1.docx]

**Supplemental Table 1. Sampling dates for arthropod collections and vegetation metrics (baregroud and grass and sagebrush ht.), number of weeks sampled, and total catch from 26 sites sampled during the 2012-2015 field seasons north of Lavina, MT.**

| Year | Treatment | Pasture/Location (Replicate)*^a^* | Arthropod and Vegetation Collection Dates*^b^* | No. Weeks Sampled | Total Catch*^c^* |
| --- | --- | --- | --- | --- | --- |
| 2012 | Deferred | 1 | 6, 21, 27 June; 5, 12 July | 5 | 1252 |
|  |  | 2 | 6, 21, 27 June; 5, 12 July | 5 | 1396 |
|  |  | 3 | 6, 21, 27 June; 5, 12 July | 5 | 847 |
|  | Grazed | 1 | 6, 21, 27 June; 5, 12 July | 5 | 538 |
|  |  | 2 | 6, 21, 27 June; 5, 12 July | 5 | 834 |
|  |  | 3 | 6, 21, 27 June; 5, 12 July | 5 | 934 |
| 2013 | Deferred | 1 | 28 May; 6, 12, 19, 27 June; 2, 10, 17 July | 8 | 1197 |
|  |  | 2 | 28 May; 6, 12, 19, 27 June; 2, 10, 17 July | 8 | 1533 |
|  |  | 3 | 28 May; 6, 12, 19, 27 June; 2, 10, 17 July | 8 | 1023 |
|  | Grazed | 1 | 28 May; 6, 12, 19, 27 June; 2, 10, 17 July | 8 | 416 |
|  |  | 2 | 28 May; 6, 12, 19, 27 June; 2, 10, 17 July | 8 | 1108 |
|  |  | 3 | 28 May; 6, 12, 19, 27 June; 2, 10, 17 July | 8 | 973 |
| 2014 | Deferred | 1 | 28 May; 3, 9, 17, 24 June; 1 July | 6 | 1695 |
|  |  | 2 | 28 May; 3, 9, 17, 24 June; 1 July | 6 | 854 |
|  |  | 3 | 28 May; 3, 9, 17, 24 June; 1 July | 6 | 1624 |
|  |  | 4 | 28 May; 3, 9, 17, 24 June; 1 July | 6 | 1314 |
|  | Grazed | 1 | 28 May; 3, 9, 17, 24 June; 1 July | 6 | 1359 |
|  |  | 2 | 28 May; 3, 9, 17, 24 June; 1 July | 6 | 1134 |
|  |  | 3 | 28 May; 3, 9, 17, 24 June; 1 July | 6 | 1972 |
|  |  | 4 | 28 May; 3, 9, 17, 24 June; 1 July | 6 | 2150 |
|  | Idle | 1 | 9, 17, 24 June;1, 8, 16 July | 6 | 2550 |
|  |  | 2 | 9, 17, 24 June;1, 8, 16 July | 6 | 2251 |
|  |  | 3 | 9, 17, 24 June;1, 8, 16 July | 6 | 2375 |
| 2015 | Idle | 1 | 27 May; 3, 10, 17, 24 June; 1 July | 6 | 3101 |
|  |  | 2 | 27 May; 3, 10, 17, 24 June; 1 July | 6 | 1605 |
| Year | Treatment | Pasture/Location (Replicate)*^a^* | Arthropod and Vegetation Collection Dates*^b^* | No. Weeks Sampled | Total Catch*^c^* |
|  |  | 3 | 27 May; 3, 10, 17, 24 June; 1 July | 6 | 1782 |

^a^Deferred and Grazed replicates equate to individual pastures (i.e., experimental unit) while Idle replicates equate to psuedoreplicated locations within the approximately 1,245 ha Lake Mason National Wildlife refuge lower unit.

*^b^*Arthropods were collected and pooled weekly from 10, nine-cm diameter; 0.5-L plastic cups (Solo Cup Company, Lake Forest, IL) in each replicate. Bare ground (%), grass height (cm), and sagebrush height (cm) were also collected weekly at each replicate by placing a 0.5 m^2^ metal ring (n=10) 2 m apart along a random compass bearing.

*^c^*Total activity-density from 54 arthropod families collected at 26 locations during 2012-2015 field seasons.
